# A Mouse Model for Studying Viscerotropic Disease Caused by Yellow Fever Virus Infection

**DOI:** 10.1371/journal.ppat.1000614

**Published:** 2009-10-09

**Authors:** Kathryn C. Meier, Christina L. Gardner, Mikhail V. Khoretonenko, William B. Klimstra, Kate D. Ryman

**Affiliations:** Department of Microbiology & Immunology and Center for Molecular & Tumor Virology, Louisiana State University Health Sciences Center, Shreveport, Louisiana, United States of America; University of North Carolina, United States of America

## Abstract

Mosquito-borne yellow fever virus (YFV) causes highly lethal, viscerotropic disease in humans and non-human primates. Despite the availability of efficacious live-attenuated vaccine strains, 17D-204 and 17DD, derived by serial passage of pathogenic YFV strain Asibi, YFV continues to pose a significant threat to human health. Neither the disease caused by wild-type YFV, nor the molecular determinants of vaccine attenuation and immunogenicity, have been well characterized, in large part due to the lack of a small animal model for viscerotropic YFV infection. Here, we describe a small animal model for wild-type YFV that manifests clinical disease representative of that seen in primates without adaptation of the virus to the host, which was required for the current hamster YF model. Investigation of the role of type I interferon (IFN-α/β) in protection of mice from viscerotropic YFV infection revealed that mice deficient in the IFN-α/β receptor (A129) or the STAT1 signaling molecule (STAT129) were highly susceptible to infection and disease, succumbing within 6–7 days. Importantly, these animals developed viscerotropic disease reminiscent of human YF, instead of the encephalitic signs typically observed in mice. Rapid viremic dissemination and extensive replication in visceral organs, spleen and liver, was associated with severe pathologies in these tissues and dramatically elevated MCP-1 and IL-6 levels, suggestive of a cytokine storm. In striking contrast, infection of A129 and STAT129 mice with the 17D-204 vaccine virus was subclinical, similar to immunization in humans. Although, like wild-type YFV, 17D-204 virus amplified within regional lymph nodes and seeded a serum viremia in A129 mice, infection of visceral organs was rarely established and rapidly cleared, possibly by type II IFN-dependent mechanisms. The ability to establish systemic infection and cause viscerotropic disease in A129 mice correlated with infectivity for A129-derived, but not WT129-derived, macrophages and dendritic cells *in vitro*, suggesting a role for these cells in YFV pathogenesis. We conclude that the ability of wild-type YFV to evade and/or disable components of the IFN-α/β response may be primate-specific such that infection of mice with a functional IFN-α/β antiviral response is attenuated. Consequently, subcutaneous YFV infection of A129 mice represents a biologically relevant model for studying viscerotropic infection and disease development following wild-type virus inoculation, as well as mechanisms of 17D-204 vaccine attenuation, without a requirement for adaptation of the virus.

## Author Summary

The highly lethal viral hemorrhagic fever caused by the mosquito-borne yellow fever virus (YFV) affects hundreds of thousands of people annually, despite the availability of the live-attenuated 17D vaccine, developed in the 1930s by serial passage of wild-type strain Asibi. Our understanding of the molecular mechanisms controlling YFV virulence and pathogenesis, and attenuation of the 17D vaccine virus, is limited, with progress greatly impeded by the lack of a small animal model reproducing human disease. Here, we demonstrate that the wild-type Asibi virus and 17D-204 vaccine substrain are readily distinguishable on the basis of morbidity, mortality and pathogenesis when inoculated subcutaneously into mice with deficiencies in their type I interferon (IFN-α/β) antiviral response. In these animals, Asibi virus infection exhibits pronounced viscerotropism, causing severe histopathology in liver and spleen, correlated with dysregulated cytokine induction, emulating hallmark features of human YF. Importantly, 17D-204 virus infection of these mice is rapidly self-limiting and subclinical as in human vaccinees. This newly developed YF disease model can be exploited to identify determinants of YFV virulence and reveal molecular mechanisms that control the virus/host interaction, providing a framework for rational design of acute phase therapeutics and improved vaccines for protection against YFV and other flaviviruses.

## Introduction

Yellow fever virus (YFV) is the prototypic member of the genus *Flavivirus*, family *Flaviviridae*; a group of arthropod-borne, positive-sense RNA viruses which also holds dengue (DENV), West Nile and Japanese encephalitis viruses [1]. YFV is endemic to tropical Central and South America and sub-Saharan Africa where the virus is mosquito-vectored between non-human primate (NHP) reservoir hosts. Introduction into human populations results in over 200,000 cases of yellow fever (YF) annually, with approximately 30,000 deaths [2]. Suspension of mosquito abatement programs, decreasing vaccination coverage and recent problems with vaccine safety and availability [3] have combined to make YFV an emerging threat within the endemic zone and beyond, in the southern United States, East Africa and previously unaffected regions of South and Central America.

In humans, YF is a pansystemic viral sepsis with 20–50% lethality, distinguishable from other viral hemorrhagic fevers (VHF) by the appearance of hepatitis and jaundice [4]. Early “flu-like” symptoms are followed by a brief period of remission, after which some infections resolve without further complications: however, illness may reappear in more severe form with high fever, vomiting and epigastric pain. Effacement of the YFV-infected liver is associated with hepatocyte apoptosis [5] and severe, diffuse steatosis [6], disproportionate to the mild, mononuclear inflammatory infiltrate [7]. Lymphoid tissues, particularly the spleen, also suffer acute damage coincident with the appearance of large, mononuclear cells and destruction of follicles [6]. Hepatic-induced disseminated intravascular coagulation (DIC) produces severe hemorrhagic manifestations. Late central nervous system (CNS) manifestations, with confusion, seizure and coma, combined with multiple organ failure, presage death which typically follows within 7–10 days of symptom onset [4]. High elevation of pro- and anti-inflammatory cytokines, particularly MCP-1, TNF-α and IL-6, during acute YF is strongly correlated with hemorrhagic symptoms and fatal outcome [8], suggesting a contribution to disease. The source of the cytokine cascade is unknown, although it is believed that hepatocytes [5], endothelial cells [9] and/or activated macrophages [4],[10] may be involved and immune clearance attempts may exacerbate viral pathogenesis [4].

Treatment of YF is limited to symptomatic and supportive care, as no specific antiviral drug is available [4]. Instead, our defense against YFV relies primarily upon vaccination with the live-attenuated YFV 17D substrains, 17D-204 and 17DD. To produce the progenitor of modern-day 17D vaccines, wild-type Asibi strain was empirically attenuated by serial passage in cultured cells [11]. Both the 17D-204 and 17DD vaccine substrains are considered safe and highly efficacious, eliciting antibody-mediated, protective immunity [3]. However, recent increases in the occurrence of fatal vaccine-associated viscerotropic disease (YEL-AVD) have raised concerns for the vaccine's future [12]. Recently, several ground-breaking studies have been published that begin to elucidate immunologic correlates of protection following 17D immunization [13]–[16] and disease in cases of YEL-AVD [17],[18].

Our understanding of the mechanisms underlying the virulence and pathogenesis of wild-type YFV, and the attenuation and immunogenicity of the vaccine, is surprisingly limited given that YFV was isolated in 1927 [11]. Existing knowledge comes primarily from natural human infection [5],[7],[8],[10],[19],[20] and NHP models of viscerotropic disease [6],[21], as small animal models have been lacking that manifest clinical signs consistent with viscerotropism, acute hepatitis and hemorrhagic fever [4]. Regardless of inoculation route, wild-type and vaccine strains of YFV cause encephalitic disease in mice, without apparent viscerotropism and, therefore, lack relevance to human infection [4], [22]–[26]. Golden hamsters develop viscerotropic disease more closely resembling human YF, but virulence must be acquired by serial passage of wild-type YFV *in vivo* [27]–[30], limiting the application of this model to the study of YFV pathogenesis. Herein, we describe our efforts to improve upon the existing small animal YFV disease models.

Wild-type YFV likely possesses mechanisms to overcome or circumvent innate immunity including type I (IFN-α/β) and/or type II (IFN-γ) interferon responses in human and NHP hosts, similar to other flaviviruses [31]–[34]. Reasoning that this ability might be primate-specific, we hypothesized that resistance of mice to fatal viscerotropic YFV infection is mediated by IFNs as it appears to be for DENV [35]–[37]. We have investigated the role of IFN-α/β *versus* IFN-γ in protection of mice against viscerotropic YFV infection. Our data suggest that: i) wild-type YFV has little or no ability to antagonize/evade the antiviral activity of IFN-α/β in mice, since the type I IFN response provides absolute defense; ii) evasion/antagonism of IFN-α/β antiviral activity may be pivotal for virulence/disease in primate hosts, but may be species-specific; and iii) the attenuated phenotype exhibited by 17D-204 virus cannot be primarily attributed to IFN-α/β sensitivity since 17D-204 virus remains attenuated in the absence of IFN-α/β signaling. By eliminating IFN-α/β signaling in mice, we have produced a model of wild-type YFV infection in which the virus causes viscerotropic disease and fatality following subcutaneous inoculation, but the vaccine strain remains attenuated.

## Materials and Methods

### Cell lines

Human Huh7 hepatocytes were maintained in Dulbecco's modified Eagle's medium (DMEM), supplemented with 10% fetal bovine serum (FBS), 0.29 mg/ml L-glutamine, 100 U/ml penicillin and 0.05 mg/ml streptomycin (37°C; 5% CO2).

### Virus stocks

Low-passage stocks of wild-type YFV strains Asibi and Angola73, and the live-attenuated 17D-204 vaccine strain were generously provided by Dr. Alan Barrett (University of Texas Medical Branch, Galveston, TX). Since isolation the Asibi virus strain was passed four times in monkeys, twice in suckling mice and five times in Vero cells. The Angola73 virus was isolated during an epidemic outbreak in Angola in 1973, and has subsequently been passed three times in suckling mice and three times in Vero cells. Both virus stocks were amplified once on Vero cells for these studies. Virus particles were harvested from cell culture supernatants when cytopathic effect was evident in the culture, clarified by centrifugation and stored at −80°C in single-use aliquots. Infectious virus titers were determined by plaque assay on Huh7 cells, expressed as plaque forming units (PFU)/mL.

### Mice

Mice deficient in receptors for IFN-α/β (A129) or IFN-γ (G129), both IFN-α/β and IFN-γ receptors (AG129; [38]) or for the STAT1 signaling molecule (STAT129; [39]) and congenic control mice (WT129) were bred under specific pathogen-free conditions. At 3–4 weeks old randomized male and female mice were transferred to the ABSL-3 facility for infection. All procedures were carried out in accordance with LSUHSC-S Institutional Animal Care and Use Committee guidelines adhering to protocols approved by the committee.

### Mortality and pathogenesis studies

For morbidity/mortality studies, virus inocula containing 10^4^ PFU of YFV Asibi, Angola73 or 17D-204 in 10 µL (i.e., 1×10^6^ PFU/mL) were administered to groups of at least four mice, subcutaneously in both rear footpads. Mock-infected mice received 10 µL PBS-1% DCS. Mice were observed at 12 h intervals and weighed daily. Average survival time (AST), percent mortality and weight changes were calculated.

For virus and cytokine titrations, groups of three mice per treatment were euthanized by isofluorane-overdose, blood was collected by cardiac puncture and serum was separated using microtainer tubes (Becton-Dickinson). Mice were perfused with PBS-1% DCS for 10 min at 7 mL/min to flush blood-borne virus. Tissues were homogenized in PBS-1% DCS and clarified by centrifugation (13,000 g, 15 min, 4°C). Serum and tissue homogenates were assayed for virus by Huh7 plaque assay. For histology, three mice per treatment were euthanized, perfused with 4% paraformaldehyde (PFA) in PBS (pH 7.4) and fixed in 4% PFA (pH 7.4). Paraffin-embedded, hematoxylin and eosin (H&E)-stained sections were viewed by light microscopy.

### Cytokine and chemokine analyses

To measure MCP-1 and IL-6 levels, serum was analyzed by X-Plex Cytokine Bead Array (BioPlex, BioRad) according to manufacturer's instructions. Briefly, serum was diluted 1∶4 in serum diluent (BioRad) and incubated with cytokine-specific antibody conjugated beads. Bead-bound cytokine molecules were detected and quantified using biotinylated, cytokine-specific antibody and streptavidin-PE. Reacted beads were fixed in 4% PFA to inactivate residual virus, washed three times and resuspended in assay buffer before analysis. Data were collected using flow cytometry-based BioPlex Suspension Array and BioPlex Manager software calculated cytokine concentrations based on a standard curve.

### Generation and infection of primary bone marrow-derived DC and macrophage cultures

Primary bone marrow-derived DCs and macrophages were generated as described previously [40]. Briefly, 8–12 week-old mice were euthanized and bone marrow was aspirated from femur and tibia bone shafts. After straining and pelleting, bone marrow cells were resuspended in DC-competent medium (cRPMI 1640 supplemented with 10% FBS, 0.29 mg/mL L-glutamine, 100 U/mL penicillin and 0.05 mg/mL streptomycin, 10 ng/mL GM-CSF and 10 ng/mL IL-4) and incubated for 1 h (37°C; 5% CO2). Non-adherent cells were removed and maintained in DC-competent medium. Adherent cells were maintained in macrophage-competent medium (DMEM supplemented with 10% FBS, 0.29 mg/mL L-glutamine, 100 U/mL penicillin and 0.05 mg/mL streptomycin, 20% L929-conditioned cell supernatant). Prior to infection, non-adherent DCs were collected from supernatants and adherent macrophages were scraped from plates. Cells were washed, counted and seeded in V-bottom 96-well plates. Cells were incubated with virus for 1 h, washed three times with PBS-1% DCS and transferred to 24-well cell culture plates in 1 mL appropriate growth medium. Samples were collected over the course of infection and titered by Huh7 plaque assay.

### Statistical analyses

All data were analyzed with GraphPad Prism software (Graphpad Software, Inc). For survival analysis, Kaplan-Meier survival curves were analyzed by the log-rank test. An unpaired Student's t-test was used to determine significant differences in virus titers and cytokine levels.

## Results

### Wild-type YFV infection is fatal for type I IFN-deficient mice, while live-attenuated 17D-204 vaccine strain is avirulent

**Figure 1:**
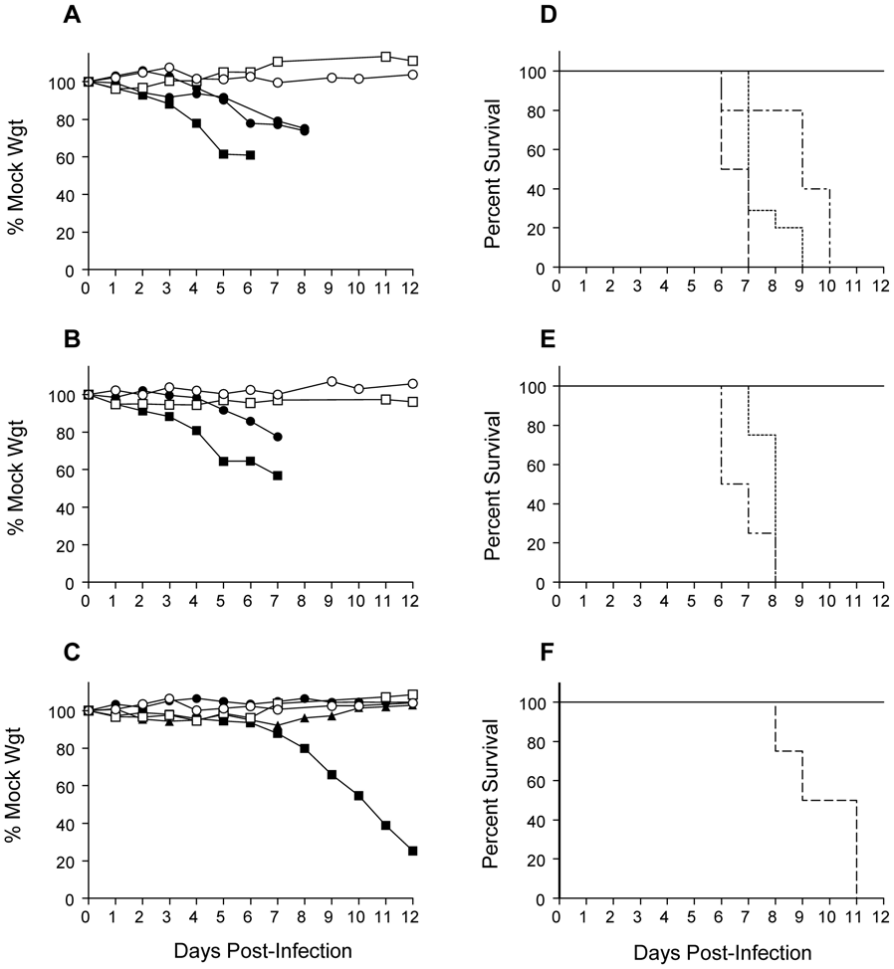
Virulence of wild-type YFV in mice is IFN-α/β-dependent while attenuation of live-attenuated 17D-204 virus is not. Adult WT129 (solid line; open circle), G129 (solid line; open square), A129 (dotted line; closed circle), AG129 (dashed line; closed square) and STAT129 (dot-dash line; closed triangle) mice were subcutaneously inoculated in each rear footpad with 10^4^ PFU of wild-type YFV strains, Asibi (A and D) or Angola73 (B and E) or with 17D-204 vaccine virus (C and F). Changes in weight from mock-infected counterparts were calculated daily for (A) Asibi virus-infected, (B) Angola73 virus-infected or (C) 17D-204 virus-infected mice as an indicator of morbidity. Percent survivals of (D) Asibi virus-infected, (E) Angola73 virus-infected and (F) 17D-204 virus-infected mice were calculated each day and are presented as Kaplan-Meier survival curves. Datum points are n≥4 and data are representative of at least two separate experiments.

Groups of 3–4 week-old mice lacking receptors for IFN-α/β (A129), IFN-γ (G129), both IFN receptors (AG129) or lacking the STAT1 signaling protein (STAT129) were inoculated subcutaneously in each rear footpad with 10^4^ PFU of wild-type YFV strains, Asibi or Angola73. Resulting morbidity and mortality were compared to infection of WT129 animals (Fig. 1 and summarized in Table 1). As expected, adult WT129 mice were disease-resistant to either wild-type YFV strain, exhibiting neither morbidity, nor mortality. Mice lacking IFN-γ receptor-dependent responses were no more susceptible to disease than controls (Fig. 1B and C) suggestive that IFN-γ was not required for protection against virulent YFV infection.

**Table 1:** Susceptibility of mice to subcutaneously inoculated Asibi and Angola73 wild-type or 17D-204 vaccine strains of yellow fever virus.

		WT129	G129	A129	AG129	STAT129
**Asibi virus**	% mortality (dead/total)	0% (0/4)	0% (0/4)	100% (7/7)	100% (4/4)	100% (5/5)
	AST±SD (d)^1^	-2	-	6.4±0.8	5.5±0.0	7.8±1.6
**Angola73 virus**	% mortality (dead/total)	0% (0/4)	0% (0/4)	100% (4/4)	100% (4/4)	ND^3^
	AST±SD (d)	-	-	6.8±0.5	5.8±1.0	ND
**17D-204 virus**	% mortality (dead/total)	0% (0/4)	0% (0/4)	0% (0/7)	100% (4/4)	0% (0/4)
	AST±SD (d)	-	-	-	10.1±1.4	-

1 AST±SD (d): average survival time±standard deviation of the mean (days).

2 - : not applicable.

3 ND: Not done.

In striking contrast, subcutaneous inoculation of mice deficient in components of type I IFN signaling pathways with wild-type Asibi or Angola73 viruses caused rapidly fatal infection (Fig. 1D and E). A129 mice exhibited signs of illness by 3–4 d p.i. with either virus, manifest as weight loss (Fig. 1A and B), lethargy, piloerection and hunched posture, with generalized cachexia and noticeable swelling at the inoculation site (not observed in WT129 animals). Animals deteriorated rapidly, becoming immobile and weak, and were typically euthanized 7–8 d p.i. Asibi or Angola73 virus-infected AG129 mice typically developed disease and died more rapidly than A129 counterparts suggestive that IFN-γ antiviral activity plays a compensatory or subordinate role to IFN-α/β. Furthermore, deficiency in STAT1-dependent IFN signaling did not render mice as susceptible as the combined absence of IFN-α/β and IFN-γ receptors or the IFN-α/β receptor only, indicating a residual STAT1-independent antiviral response, mediated by IFN-α/β and/or IFN-γ. Importantly, none of the Asibi or Angola73 virus-infected A129, G129 or STAT129 mice developed any clinical signs typically associated with neurologic disease such as ataxia, paresis or hind-limb paralysis. This differs dramatically from existing small animal models of fatal wild-type YFV infection, in which encephalitic paralysis is the major clinical sign following intracerebral or peripheral inoculation [4].

In contrast to the wild-type viruses, subcutaneous inoculation of A129 or STAT129 mice with the 17D-204 vaccine strain caused no clinical signs of disease or mortality (Fig. 1C and F). The lack of 17D-204 virulence in these type I IFN-deficient mouse strains suggests that attenuation of human virulence may not be solely a result of altered sensitivity to IFN-α/β activity. Interestingly, 17D-204 virus caused a uniformly fatal infection in AG129 mice, consistent with the findings of Lee & Lobigs [41]. These data signify that IFN-γ is required to attenuate the vaccine in the absence of the type I IFN response, likely in a largely STAT1-independent manner as STAT129 mice survived without signs of disease. However, the AST of 17D-204 virus-infected AG129 mice was significantly longer than Asibi or Angola73 virus-infected counterparts (10.1±1.4 *versus* 5.5±0.0 days; *p<0.004*), indicating that the vaccine strain remained relatively attenuated. Furthermore, 17D-204 virus-infected AG129 mice developed signs of neurologic disease with paresis and paralysis, not seen in wild-type YFV-infected counterparts, suggestive that neuroinvasion/neurovirulence of the vaccine strain in the absence of IFN-α/β and IFN-γ responses contributes to mortality.

### Control of wild-type YFV replication and dissemination is dramatically reduced in type I IFN-deficient mice

**Figure 2:**
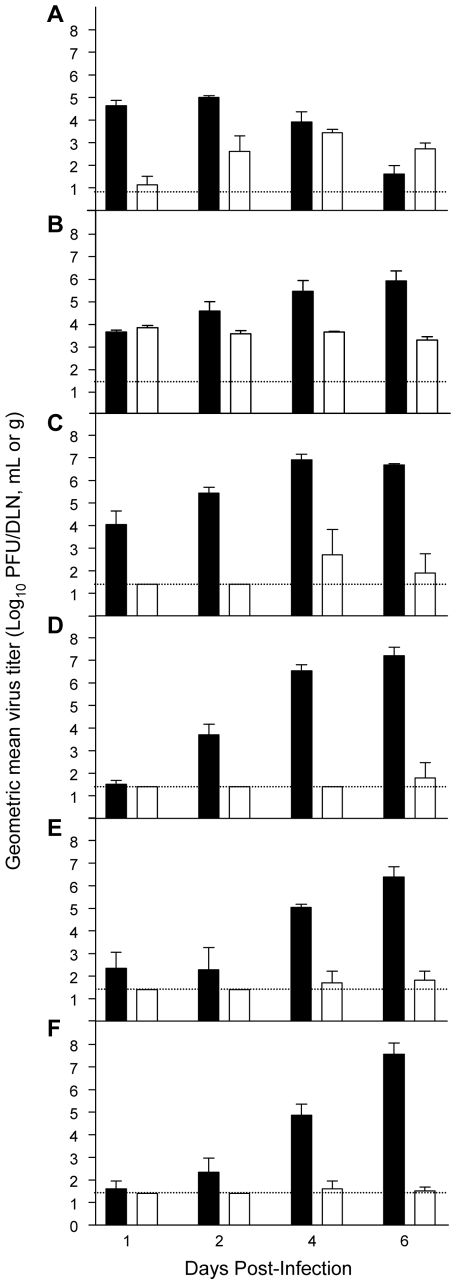
Wild-type Asibi virus exhibits viscerotropism in type I IFN-deficient mice, whereas 17D-204 virus does not. WT129 (data not shown) and A129 mice were subcutaneously inoculated with 10^4^ PFU of Asibi (black bar) or 17D-204 (white bar) virus in each rear footpad. Viral titers in individual, perfused tissues are expressed as log_10_ PFU/ml of serum, PFU/draining lymph node (DLN) or PFU/g of other tissues: (A) DLN; (B) serum; (C) spleen; (D) liver; (E) bone marrow; and (F) brain. Dotted lines represent the lower limit of detection for the plaque assay. Datum points are *n = 3±SD* for all data sets.

Since IFN-α/β controls replication and dissemination of many viruses, including other flaviviruses [42]–[46], we reasoned that increased susceptibility of type I IFN-deficient mice to wild-type YFV infection would correspond to higher virus titers and potentially altered tropism compared with 17D-204 virus infection or wild-type YFV infection of disease-resistant WT129 mice. To test this hypothesis, we focused upon the YF disease in mice lacking the IFN-α/β receptor. WT129 and A129 mice were inoculated subcutaneously with 10^4^ PFU of Asibi or 17D-204 virus and viral loads in serum and PBS-perfused tissues were measured (Fig. 2). In parallel samples harvested from WT129 mice, replication of neither Asibi, nor 17D-204, virus was detectable in any tissue or at any time-point analyzed (data not shown).

Regional lymph nodes draining the inoculation site (DLN) are an early amplification site for several other flaviviruses, important for seeding the primary serum viremia and dissemination [47]. In the absence of IFN-α/β-mediated antiviral responses, Asibi virus amplified extensively in the popliteal DLN (Fig. 2A) and serum viremia was detectable within 1 d p.i., increasing over the course of infection (Fig. 2B). Asibi virus disseminated to the visceral organs of A129 mice, represented by spleen and liver, within 1 d p.i., reaching sustained levels of >10^7^ PFU/g by 4 d p.i. (Fig. 2C and D, respectively) and was also detected in bone aspirate (Fig. 2E). Titration of whole brain homogenates revealed detectable virus by 4 d p.i. (Fig. 2F). Due to the absence of neurologic signs of disease, however, we speculate that replication is predominately restricted to the meninges and/or endothelial cells, sparing the brain parenchyma, similar to recent findings with another non-encephalitic arbovirus, chikungunya virus [48]. Regardless, CNS replication is a late event, occurring after the onset of clinical disease signs.

Consistent with morbidity/mortality results, 17D-204 virus replication and dissemination were profoundly restricted in A129 mice (Fig. 2). While the vaccine virus was able to replicate in the DLN in the absence of IFN-α/β responses, amplification was delayed and reduced in comparison to parental Asibi virus (Fig. 2A), peaking at almost 100-fold lower levels. Furthermore, although the presence of a low-level serum viremia suggested that 17D-204 virus disseminated systemically (Fig. 2B), virus replication was only sporadically detected beyond the DLN (Fig. 2C–F), consistent with the lack of morbidity or mortality observed in 17D-204 virus-infected A129 animals.

### Wild-type YFV infection of type I IFN-deficient mice induces proinflammatory cytokine release

**Figure 3:**
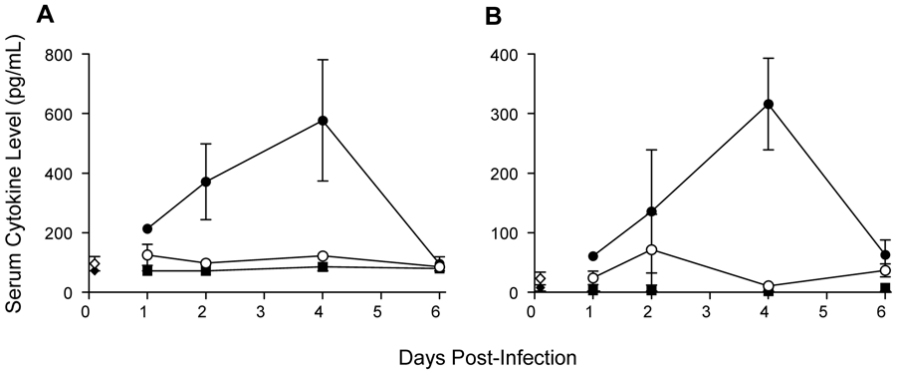
Cytokine levels are elevated in Asibi virus-infected A129 mice. Serum levels of (A) IL-6 and (B) MCP-1 were measured in serum collected from mock-infected WT129 (open diamond) and A129 (closed diamond) mice, Asibi virus-infected WT129 (open circle) and A129 (closed circle) mice and 17D-204 virus-infected A129 mice (closed square) by cytokine bead array analysis (BioRad BioPlex Assay). Data are expressed as concentration of cytokine in pg/mL serum where datum points are *n = 3±SD*. Mock values were generated from pooled data over the course of infection.

**Figure 4:**
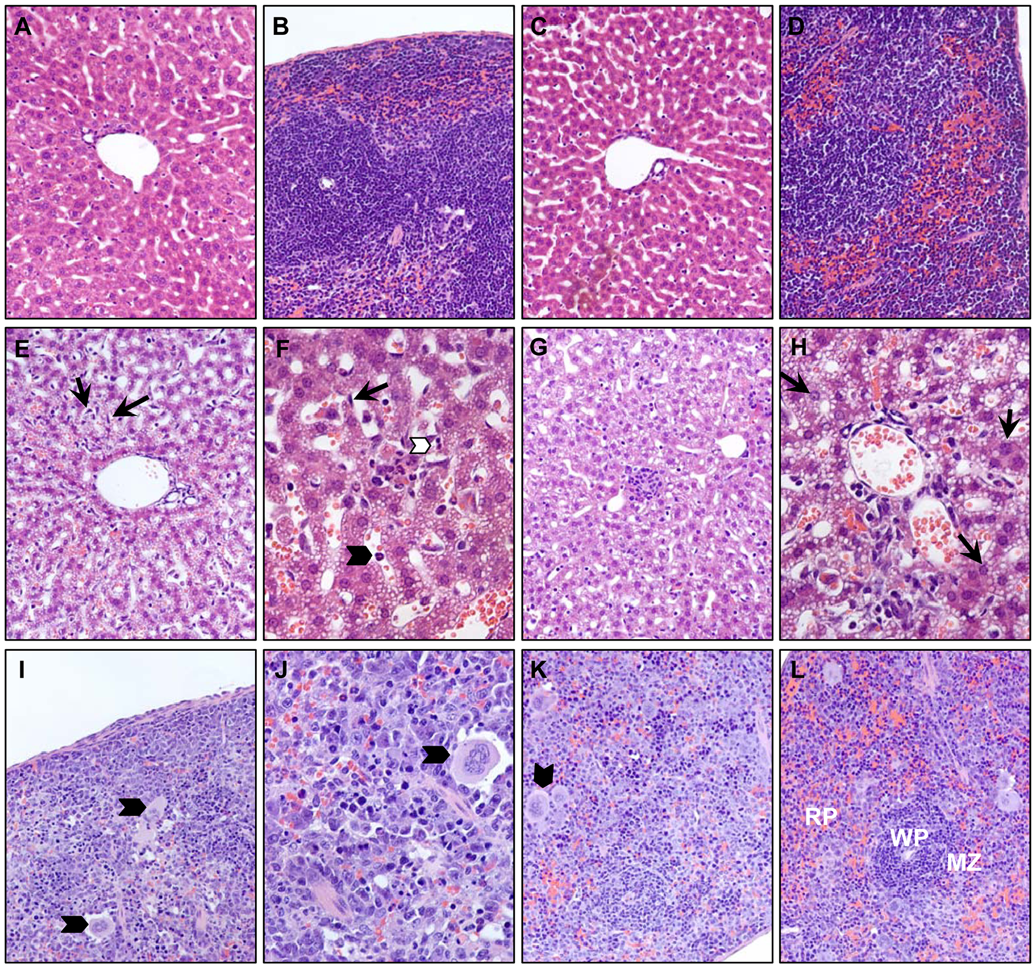
Pathologic changes were evident in liver and spleen of Asibi virus-infected A129 mice. A129 mice were mock-infected or inoculated with 10^4^ PFU of Asibi or 17D-204 viruses in each rear footpad. H&E-stained sections of liver and spleen are presented. Liver (A) and spleen (B) sections from mock-infected A129 animals were indistinguishable from 17D-204-infected liver (C) and spleen (D) sections, 4 d p.i. (E and F) Liver sections from Asibi virus-infected A129 mice 4 d p.i., showing diffuse inflammatory infiltrates, well-developed fatty acid steatosis involving the majority of the lobular area. Note prominent Küpffer cells (black arrows) and some accumulation of inflammatory cells in hepatic sinusoids (arrow heads). *Original magnification: (E) 200×; (F) 400×*. (G) Asibi virus-infected liver 6 d p.i., showing and a focus of spotty necrosis (center of field) surrounded by mononuclear inflammatory cells and higher magnification (H) showing more severe inflammatory cell infiltration surrounding portal triad and hepatocytes with intranuclear viral inclusion bodies (black arrows). *Original magnification: (G) 200×; (H) 400×*. (I and J) Spleen sections from Asibi virus-infected A129 mice 4 d p.i. and (K and L) 6 d p.i., showing diminishing marginal zone (MZ) and white pulp lymphoid follicles (WP), increasing number of splenic macrophages (arrow heads) and widely-distributed cells with pyknotic nuclei, morphologically consistent with apoptotic death. *Original magnification: (I, K, L) 200×; (J) 400×*.

In human YF and YEL-AVD, fatal hemorrhagic disease is characterized by a systemic inflammatory response or “cytokine storm,” with profoundly elevated serum levels of proinflammatory cytokines and chemokines [8],[18],[49]. To determine whether or not fatal Asibi virus infection of A129 mice similarly dysregulated the inflammatory cytokine response, key inflammatory molecules were measured in serum from Asibi virus-infected A129 and WT129 mice, and from 17D-204 virus-infected A129 mice (Fig. 3). ter Meulen *et al.* [8] demonstrated that, amongst a panel of cytokines, levels of IL–6, MCP-1 and TNF-α were all statistically significantly higher in patients with fatal, hemorrhagic YF than in those with non-fatal YF. Consequently, we selected these cytokines for analysis in our studies. Unfortunately, due to the necessity for inactivation of virus in samples prior to analysis and the instability of the TNF-α protein, neither ELISA nor BioPlex assays were successful for this cytokine. Coincident with the onset of morbidity 4 d p.i., a hyper-inflammatory cytokine response was observed in Asibi virus-infected A129 mice, with high levels of IL-6 (Fig. 4A) and MCP-1 (Fig. 3B). Correlated with self-limiting, subclinical infection, proinflammatory cytokine levels in Asibi virus-infected WT129 mice and 17D-204 virus-infected A129 mice remained around base-line throughout, consistent with the lack of morbidity/mortality.

### Wild-type YFV causes severe pathology in visceral organs of type I IFN-deficient mice

Gross examination of organs from Asibi virus-infected A129 mice revealed evidence of hepatosplenomegaly by 4 d p.i., with the livers appearing discolored and icteric, whereas organs from 17D-204 virus-infected A129 mice and Asibi virus-infected WT129 mice appeared normal (data not shown). Tissues harvested from Asibi virus-infected WT129 mice (data not shown) or from 17D-204 virus-infected A129 mice were histologically indistinguishable from mock-infected controls (Fig. 4A–D). However, severe histopathologic changes were observed in visceral organs from Asibi virus-infected A129 mice. At 4 d p.i., coincident with disease onset and peak cytokine induction, lobular disarray of the infected liver was observed with well-developed microvesicular fatty acid steatosis involving much of the lobule parenchyma (Fig. 4E and F). Spotty hepatocyte death was associated with clusters of infiltrating inflammatory cells and, moreover, vascular congestion and fibrin deposition were evident. By 6 d p.i., when mice were moribund, focal inflammation of the portal triad was observed, while the inflammatory infiltration and steatosis remained essentially the same (Fig. 4G and H). Histopathologic changes in the spleens of Asibi virus-infected A129 animals consisted of dramatic follicular depletion accompanied by lymphocytic necrosis within 4 d p.i. with loss of follicular organization throughout, accompanied by an infiltration of macrophages into white and particularly red pulp regions (Fig. 4I and J). Similar, but more severe histopathology was observed by 6 d p.i. (Fig. 4K and L).

### Infectivity for DCs and macrophages is IFN-α/β-dependent and correlates with disease

**Figure 5:**
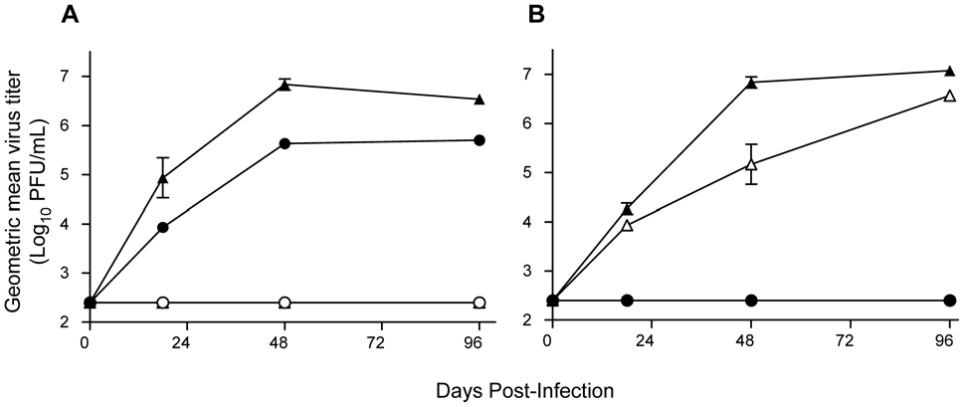
YFV strains Asibi and 17D exhibit differential infectivity for murine DCs and macrophages *in vitro*. Cultures of primary bone marrow-derived DCs (A) and macrophages (B) generated from WT129 (circle) or A129 (triangle) mice were infected with wild-type Asibi virus (closed) or live-attenuated 17D-204 virus (open) at MOI = 0.1 PFU/cell. Viral replication was measured by plaque assay titration of progeny virions in culture supernatants. Data are expressed as log_10_ PFU/mL where datum points are *n = 3±SD*. These data were reproducible in three separate experiments.

Current models of YFV pathogenesis propose that infection of DCs and/or macrophages may be a crucial early event, in which the virus exploits migratory properties of activated cells to effect viremic dissemination [4], similar to other arboviruses. Furthermore, these cells may contribute to pathogenesis and cytokine induction [4], [50]–[52]. Accordingly, we have assessed the permissiveness of primary, bone marrow-derived DCs and macrophages to infection with Asibi and 17D-204 viruses *in vitro* (Fig. 5). DCs derived from either WT129 or A129 mice supported the replication of Asibi virus (Fig. 5A), with progeny virions detectable within 18 h p.i. and rising to high titers. In contrast, DCs were virtually refractory to 17D-204 virus infection, regardless of their IFN-α/β competence (Fig. 5A), although very low levels of progeny 17D-204 virus were detectable in some experiments. Few progeny virions were released from WT129-derived macrophages exposed to either Asibi or 17D-204 virus, but refractivity of macrophages was IFN-α/β signaling-dependent, as both Asibi and 17D-204 viruses amplified efficiently in A129-derived macrophages (Fig. 5B). Thus, it appears that productive infection of both DCs and macrophages correlates with fatal disease, since Asibi virus replicates in both DCs and macrophages generated from A129 mice.

## Discussion

Molecular mechanisms of YFV pathogenesis, virulence and attenuation are not well understood, impeded by the lack of an appropriate small animal model of viscerotropic YF disease caused by the wild-type virus without the need for adaptation [4]. To date, attempts to develop a mouse model of viscerotropic YFV infection have been unsuccessful, with infected animals typically exhibiting signs of encephalitic disease. To gauge the extent to which IFN-mediated antiviral responses control the virulence of YFV in mice, we evaluated YFV wild-type and vaccine infection in animals lacking important components of the IFN-α/β and/or IFN-γ signaling pathways. Based upon our findings, we posit that YFV infection of IFN-α/β receptor-deficient mice provides a valuable small animal model, exhibiting key features of fatal human YF following wild-type virus infection. Furthermore, subcutaneous infection of A129 mice readily distinguishes virulent wild-type YFV infection from the live-attenuated 17D-204 vaccine strain. It should be noted that comparison to the established NHP YF disease model will be important and beneficial to fully validate the A129 model.

In the absence of type I IFN responses, wild-type Asibi virus infection of A129 mice led to rapid viral dissemination, most likely by infecting and commandeering migratory DCs and/or macrophages, as described for other flaviviruses [37],[53]. Asibi virus amplified in the DLN, seeded a serum viremia and spread to other tissues, notably exhibiting viscerotropism resembling human YF [10]. In particular, the liver was a site of extensive Asibi virus replication in A129 mice and suffered extensive damage, including widespread fatty acid steatosis, mild inflammatory infiltration by lymphocytes and macrophages, as well as apoptotic/necrotic lesions, consistent with profound hepatic disease associated with human YFV infections [4],[6],[10],[19],[20]. Similar hepatotropic disease has also been observed in NHP with wild-type YFV [6],[21] and in hamster models, primarily using hamster-adapted YFV strains [28]. Our study is the first to report viscerotropism with histopathologic damage in a small animal model using native, wild-type YFV strains.

Human YF disease is associated with a profound inflammatory response in which serum concentrations of IL-6 and MCP-1, as well as other cytokines, are significantly elevated and predictive for severity of YF, distinguishing fatal hemorrhagic YF, non-fatal hemorrhagic YF and non-fatal/non-hemorrhagic YF [8]. Moreover, in cases of fatal YEL-AVD, which resemble natural YF, similar patterns of cytokine induction have been reported [18],[49]. In Asibi virus-infected A129 mice IL-6 and MCP-1 were strongly induced, peaking at 4 d p.i., coincident with the onset of disease. Perhaps significantly, during trauma-hemorrhage and sepsis, two disease etiologies with significant parallels to human YF, liver-resident Küpffer macrophages are a major source of IL-6 and MCP-1 [54]. These molecules act on immune cells leading to local and generalized inflammation and affect vascular (endothelial) function by modulating vascular nitric oxide and superoxide release and mediating vascular disorders including shock and DIC. Recent studies suggest that many factors contribute to YFV-associated tissue damage in humans, including viral replication and inflammatory cytokine mediators [7],[20], which can be further investigated in the A129 disease model prior to investigation in NHP models.

Further validating this model of YF disease, attenuated 17D-204 virus infection of A129 animals was correlated with an apparently abortive virus infection, greatly reduced viscerotropism and no elevation of proinflammatory cytokines or tissue pathology. Thus, the pathogenesis of 17D-204 virus was restricted compared with the wild-type virus even in the absence of type I IFN activity. Since 17D-204 virus infected A129-derived DCs poorly *in vitro*, the delayed appearance of 17D-204 virus in the DLN may reflect a differential cell tropism and necessity to disseminate *via* a different mechanism. Interestingly, A129-derived macrophages were permissive to 17D-204 virus infection *in vitro* and, therefore, macrophages or an alternate DC subset may serve as a vehicle for dissemination and amplification *in vivo*. The 17D-204 vaccine strain triggers activation and maturation of human DCs *in vitro via* multiple toll-like receptors [50],[51], perhaps contributing to immunogenicity in vaccinees. However, there is controversy as to whether or not these cells are productively infected by 17D-204 virus [51],[55], and their relative permissiveness to wild-type YFV infection has not been assessed *in vitro* or *in vivo*. Our data suggest that reduced ability to amplify in specific subsets of myeloid-lineage cells may be one mechanism of 17D-204 attenuation; however, further studies will be necessary to define the step(s) at which 17D-204 virus infection is restricted compared to Asibi virus, resulting in the attenuated phenotype; knowledge that will facilitate the identification of molecular determinants of attenuation and viscerotropic disease in YFV.

Finally, a central finding of this study is that YFV sensitivity to murine IFN-α/β-mediated antiviral responses is sufficient to protect against disease, while loss of the IFN response leads to inflammatory, viscerotropic disease with striking similarities to human YF and YEL-AVD. While the type I IFN response is essential for protection in the mouse model, virulent human infections occur in individuals with properly functioning IFN responses. The apparent difference in efficacy between human and murine IFN-α/β against YFV infection suggests a level of species-specificity, where the human IFN-α/β response is significantly less effective than murine in restricting YFV pathogenesis. Alternatively, YFV may have evolved mechanisms to overcome or evade IFN-α/β responses in natural primate hosts that are relatively ineffective against murine IFN-α/β responses. Interestingly, similarities to studies with DENV [35],[37],[46] and chikungunya virus [48] suggest this phenomenon might be generally applicable to mosquito-borne viruses that are restricted to primate reservoir hosts in nature and non-encephalitic. More studies are necessary to determine the differences in species specificity of IFN-α/β responses on viruses like YFV. We anticipate that future studies will better define the specific differences between infection of humans and immune-competent mice. In the meantime, although A129 mice may not perfectly model YFV infection of humans, we believe they will be valuable for studying the virulence/attenuation of YFV and developing hypotheses to be tested in focused non-human primate studies.
